# Improving Consumption and Purchases of Healthier Foods in Retail Environments: A Systematic Review

**DOI:** 10.3390/ijerph17207524

**Published:** 2020-10-16

**Authors:** Allison Karpyn, Kathleen McCallops, Henry Wolgast, Karen Glanz

**Affiliations:** 1 Center for Research in Education and Social Policy, University of Delaware, Pearson Hall, 125 Academy Street, Newark, DE 19716, USA; kamcca@udel.edu (K.M.); hnrywlg@udel.edu (H.W.); 2 Perelman School of Medicine and School of Nursing, University of Pennsylvania, 3400 Civic Center Blvd, Philadelphia, PA 19104, USA; kglanz@pennmedicine.upenn.edu

**Keywords:** food access, nutrition, healthier food, dietary behaviors, review, retail food environment, dietary intake

## Abstract

This review examines current research on manipulations of U.S. food retail environments to promote healthier food purchasing and consumption. Studies reviewed use marketing strategies defined as the 4Ps (product, price, placement, promotion) to examine results based on single- and multi-component interventions by study design, outcome, and which of the “Ps” was targeted. Nine electronic databases were searched for publications from 2010 to 2019, followed by forward and backward searches. Studies were included if the intervention was initiated by a researcher or retailer, conducted in-store, and manipulated the retail environment. Of the unique 596 studies initially identified, 64 studies met inclusion criteria. Findings show that 56 studies had at least one positive effect related to healthier food consumption or purchasing. Thirty studies used single-component interventions, while 34 were multi-component. Promotion was the most commonly utilized marketing strategy, while manipulating promotion, placement, and product was the most common for multi-component interventions. Only 14 of the 64 studies were experimental and included objective outcome data. Future research should emphasize rigorous designs and objective outcomes. Research is also needed to understand individual and additive effects of multi-component interventions on sales outcomes, substitution effects of healthy food purchases, and sustainability of impacts.

## 1. Introduction

The promotion of healthy purchasing in shopping environments is a focal point of public health and research efforts aimed at reducing obesity and improving health outcomes. In the U.S., 71.2% percent of adults and 41.0% of children ages 2–19 have overweight or obesity, a condition that increases risk for cardiovascular disease, cancer, and diabetes [1,2]. Recent examination of American diets found most Americans eat more total calories, saturated fat, salt, and added sugar than they need, and do not consume enough fruits and vegetables, and whole grain products [3]. The majority of food purchasing occurs in supermarkets, which are uniquely positioned between the consumer and food purchasing decisions [4]. In addition to providing access to food, the in-store food retail environment is recognized for its influential role in dietary outcomes [5]. In-store, food retail interventions influencing the food purchasing decisions of consumers have grown in popularity over the past 10 years. This shift is in part due to the popularity of behavioral economics as a foundation by which customers may be “nudged”, though indirect suggestions, toward healthier products [6,7]. Most commonly, research on in-store approaches is characterized by the 4Ps of marketing (product, price, promotion, and place) and approaches targeting consumer purchasing habits toward “better-for-you” products [8,9]. Such products are often lower-calorie, lower-sugar, lower-salt, or include more whole grains. Better-for-you products have been promoted in food retail settings to reach those at highest risk for diet-related disease [10].

Despite growing research, increasing recognition of the importance of marketing in the food retail environment and the popularizing practice of multi-component interventions, which manipulate more than one of the four Ps [11], there remain many unanswered questions about best practices for implementing effective in-store interventions. Food marketing and consumer behavior research is cross-disciplinary by nature, with outcomes published in outlets unique to industry, business, agriculture as well as public health, creating an aggregation challenge for practitioners.

This review seeks to update and build on prior reviews which terminate with studies published on or around 2010 [9,12,13] by analyzing U.S.-specific interventions occurring within the past 10 years with the goal of examining the extent to which contemporary manipulations of U.S. food retail environments (i.e., grocery and supermarket) specifically intended to promote healthier food purchasing and consumption are effective. Findings were synthesized and organized based on whether the intervention was a single-component intervention, which manipulates one of the four Ps, or a multi-component intervention, which manipulates more than one of the four Ps, and further broken down into the 4Ps of marketing and study design: Experimental, quasi-experimental, pre-experimental, and time series. An emphasis is placed on the marketing techniques utilized in study interventions in order to determine which strategies have been found to be most and least effective using different research designs and outcome measures.

## 2. Materials and Methods

This review used the Preferred Reporting Items for Systematic Reviews and Meta-analyses (PRISMA) guidelines [14].

### 2.1. Search Strategy

The authors used several methods to ensure a thorough and comprehensive review of the literature on in-store marketing interventions for healthy food promotion. First, a list of inclusion criteria was created to identify papers to be included in the review sample. Second, a list of key terms was created to search for studies. Third, appropriate databases were identified for the search based on the database topics. Finally, the database search was conducted to identify inclusion articles, and forward and backward searches were conducted for each inclusion article. Below are the processes used to identify studies for this review.

### 2.2. Inclusion Criteria

The studies included are original empirical research published between 2010 and 2019, in English, and from the United States. Studies were researcher- or retailor-initiated, conducted inside the retail environment, and manipulated the retail environment. Evaluations could be quantitative or mixed methods and all interventions had to include at least one of the following outcomes: (1) Purchasing-related (i.e., objective store sales data, objective food purchasing data, customer receipts, and survey self-reported purchases or expenditures, store sales, or intent to purchase), and/or (2) consumption-related (i.e., food frequency questionnaire (FFQ), 24-h dietary recall, food diary, Veggie Meter^TM^ or other biometrics, or other survey self-reported diet/consumption or intent to eat).

### 2.3. Exclusion Criteria

Interventions were excluded if they were implemented by an entity other than a researcher or retailer (e.g., price intervention at the wholesale level or front-of-pack labels initiated by a food company), if they did not occur inside the retail environment (e.g., restaurants, schools, mobile food trucks, online, and laboratory), or if they did not manipulate the retail environment (e.g., grocery store tours).

### 2.4. Search Terms and Databases

Nine databases (i.e., Academic OneFile, Business Source Premier, CAB Abstracts, Communication and Mass Media Complete, Family and Society Studies Worldwide, PsycINFO, PubMed, Sociological Abstracts, and Web of Science) from a variety of sectors (i.e., agriculture, business, communication, health, and psychology) were searched. Key terms were constructed based on three concepts: (1) Healthier food, (2) study design, and (3) setting. A variety of search terms were used to ensure articles would be included with nuanced differences in terms (e.g., healthy food vs. better-for-you) across sectors. The following key terms were used in all databases:
Healthier food“health* food*” OR “healthy eating” OR “fruit*” OR “vegetable*” OR “low* fat” OR “low* sodium” OR “low* sugar” OR “low-fat” OR “low-sodium” OR “low-sugar” OR “better for you” OR “nutritio*”Study design“intervention” OR “pilot” OR “experiment*”Setting“supermarket*” OR “grocery store*” OR “corner store*” OR “bodega*” OR “retail environment”

### 2.5. Procedure of Article Search

**Figure 1:**
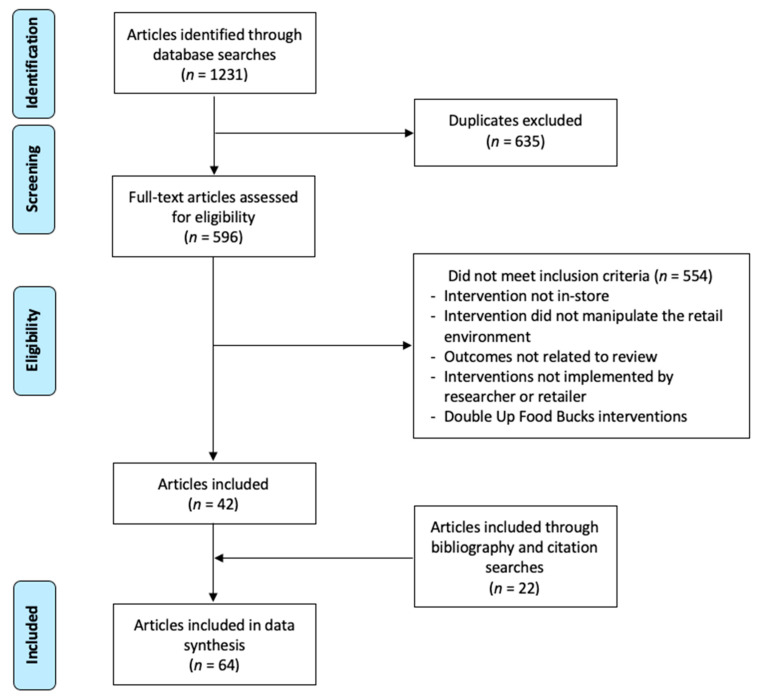
Article inclusion flow chart.

RefWorks database was used to organize all articles. The searches were conducted by two authors and yielded 1231 studies (see Figure 1). After excluding 635 duplicate articles, two authors reviewed each full-text article to determine eligibility and excluded 548 studies. This review yielded 42 articles that met all inclusion criteria. Then, citation and bibliography searches were conducted with all 42 articles identifying an additional 22 articles for a final total of 64 articles (see Table 1).

After removing duplicates, two reviewers independently screened the title, abstract, and full text of the remaining 596 articles. Reviewers discussed any differences and consulted a third reviewer, when necessary, and a consensus was reached. One reviewer conducted forward and backward searches of the included articles. Titles and then full texts were reviewed to assess eligibility. Articles were abstracted and coded independently with two coders; discrepancies were discussed until a consensus was reached. Article abstractions included participants, study design, intervention description, 4 Ps, intervention setting, duration of intervention, data collection methods, outcome variables, and key findings. Our research reviewed studies and categorized them according to the 4 Ps: Product, price, promotion, and/or placement. Examples of interventions that were classified as product included determining how many and how much variety of a product to stock. Interventions that examined price included strategies such as price reductions and coupons. Furthermore, examples of interventions classified as promotion included shelf labels, recipe cards, and taste tests, and examples of placement strategies included altering the in-store location of products, such as moving to an endcap or to eye level. Our review included an examination for biases, with a focus on research design (eliminating confounders) and measures (i.e., self-report vs. objective data). Bias was assessed using the principles laid out in the Cochrane risk of bias tool [15].

## 3. Results

### 3.1. Features of Included Articles

The primary intervention sites varied in terms of store size and included supermarkets (43.8%), corner stores (31.3%), grocery stores (26.6%), and/or convenience stores (9.4%) (see Table 1). Experimental designs accounted for about one-third (35.4%) of available studies, while the remaining were pre-experimental (33.8%), quasi-experimental (27.7%), or time series (3.1%). The most frequently used objective outcome data were store sales data (46.9%), while self-reported purchasing or expenditures was the most frequently used self-report measure (40.6%). Intervention length varied from 22 min to 3.5 years. Most studies (89%) incorporated promotion as a key component of the intervention, although efforts to address product (34%) and placement (31%) were also prominent. Relatively few interventions focused on price (16%). A total of 56 of 64 studies (87.5%) had at least one positive effect. When considering only objective measures of sales and more rigorous methods of determining dietary intake (i.e., 24 h recalls or biometric data), 100% (14 out of 14) had at least one positive effect.

### 3.2. Single- and Multi-Component Interventions

**Figure 2:**
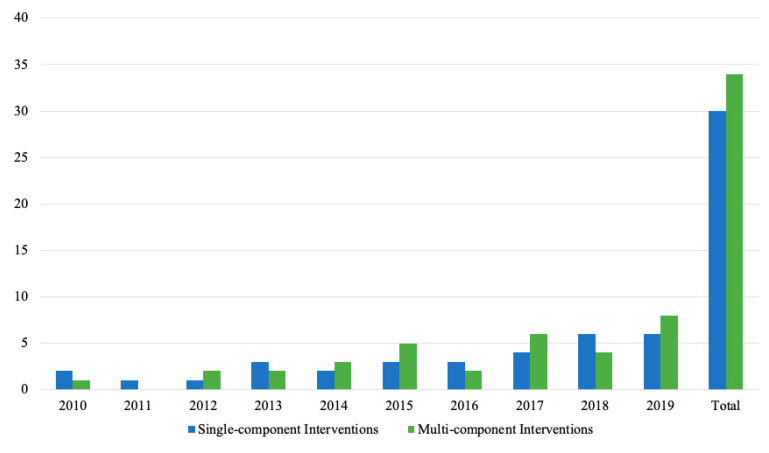
Number of single- and multi-component interventions by year.

Thirty interventions were classified as single-component interventions because they only manipulated one of the four Ps, while 34 interventions were classified as multi-component. Over the past 10 years, the number of single- and multi-component interventions have both slightly increased (see Figure 2).

### 3.3. Single-Component Interventions

Among the 30 single-component interventions, 27 had at least one positive effect on improving consumption and purchasing of healthier foods. Promotion was the most commonly utilized marketing P and was the focus of 23 studies. Overall, 1 study had mixed effects (positive + negative) [16], 5 had mixed effects (positive + null) [17,18,19,20,21], 8 had mixed effects (positive + null + negative) [22,23,24,25,26,27,28,29], 1 had negative effects [30], 2 had null effects [31,32], and 13 had positive effects [33,34,35,36,37,38,39,40,41,42,43,44,45], (see Table 2).

#### 3.3.1. Product

Of the 30 single-component interventions, only one intervention manipulated product [40]. The study utilized a pre-experimental design and found positive effects on produce sales after increasing stocking and availability of fresh produce [40].

#### 3.3.2. Placement

One study implemented a placement-only intervention [23]. This experimental study had mixed effects (positive + null + negative). Positive effects were found such that featuring healthy products in aisle endcaps increased sales of these healthy products. However, when healthy products and indulgent products were featured together in aisle endcaps, sales of indulgent products increased while healthy products did not increase [23].

#### 3.3.3. Price

Three studies implemented price-only interventions [18,29,33]. One study had mixed effects (positive + null + negative) [29], while another had mixed effects (positive + null) [18] and one study had only positive effects [33]. Both experimental studies provided a 50% discount for fruits and vegetables [18,33] and found that customers who received the discount purchased significantly more fruits and vegetables than customers who did not receive the discount [18,33]. However, one study also found no sustained effect on participants’ spending on fruits and vegetables from baseline to follow-up period [18]. In addition, one study used a pre-experimental design [29].

#### 3.3.4. Promotion

Twenty-three studies used a promotion strategy [17,19,20,21,22,24,25,26,27,28,30,31,32,34,36,37,38,39,41,42,43,44,45] as the sole intervention approach. Ten promotion interventions had positive effects [34,36,37,38,39,41,42,43,44,45], four reported mixed effects (positive + null) [17,19,20,21], six reported mixed effects (positive + null + negative) [22,24,25,26,27,28], two reported null effects [31,32], and one reported negative effects [30].

Four studies used experimental designs [17,18,22,34]. The interventions focused on nutrition shelf labeling [18], food samples [34], nutrition education [17], and a smartphone app [24]. One study found positive effects on fruit and vegetables purchases [34], while another study found mixed effects (positive + null) on food purchasing (e.g., positive effects on servings of fruit and no effect on servings of vegetables) [17]. Two studies found mixed effects (positive + null + negative) for the change in consumption and purchases of products authors classified as healthier (e.g., fruits, vegetables, and whole grains) as compared to products identified as less healthy (e.g., higher calorie products and sweets) [24] and for change in the sale of popcorn using different nutrition shelf labels [22]. One example of a study with largely positive results used a combination of shelf labels (e.g., “healthier option,” “low sodium”) in combination with education about the labels [17]. Positive effects were found such that customers purchased more servings of fruits and dark-green/bright-yellow vegetables. However, there were no significant differences between the groups on saturated fat, trans fat, and servings of vegetables [17].

Eight studies focused only on promotion, utilizing quasi-experimental designs [25,26,30,31,36,37,38,39]. Of these, four studies tested shelf labels [30,36,37,39], one examined the effectiveness of nutrition information labeling [26], one utilized a mass media campaign [25], one tested food demonstrations [38], and one examined the ability of increased stocking and promotions to sell healthy items [31]. Three found positive effects on purchases of healthier products [36,37,39] and one found positive effects regarding self-reported fruit and vegetable consumption [38]. One study had null effects on healthy food purchases and consumption, using both self-report measures and a skin carotenoid test [31]. Another study found negative effects on the demand for healthy popcorn [30]. Two studies found mixed effects (positive + null + negative) on sales of milk and the influence of caloric information on purchases [25,26].

Pre-experimental study designs reflected the majority of single-component intervention studies employing promotion [19,20,21,27,28,32,41,42,43,44,45]. Five implemented shelf labels [19,21,27,28,41], two used mass media social marketing campaigns [32,45], two implemented podcasts [20,42], one used taste tests [43], and one used a smartphone app [44].

#### 3.3.5. Other

In addition to the 4Ps, two studies did not fit into the standard 4P framework and therefore were classified as “other” [16,35]. Both utilized experimental study designs. One examined the effects of ambient music [16] and the other study analyzed effects of ambient scents [35]. Findings showed mixed effects (positive + negative) as lower volume music increased healthier purchasing patterns and higher volume music increased unhealthier purchases [16]. Additionally, findings showed positive effects when using an in-store indulgent scent (i.e., chocolate chip cookies), which led to increased purchasing of healthier foods, and decreased purchasing of unhealthy foods [35].

### 3.4. Multi-Component Interventions

Out of the 34 multi-component interventions, 13 interventions included two Ps [46,47,48,49,50,51,52,53,54,55,56,57,58], 20 interventions included three Ps [59,60,61,62,63,64,65,66,67,68,69,70,71,72,73,74,75,76,77,78], and one intervention included all four Ps [79]. All of the multi-component interventions included promotion. Overall, 8 had positive effects [57,58,65,66,71,73,75,76], 2 studies had mixed effects (positive + negative) [68,78], 13 had mixed effects (positive + null) [46,47,49,51,52,53,56,59,60,61,62,64,72], 4 had mixed effects, (positive + null + negative) [50,55,63,77], and 7 had null effects [48,54,67,69,70,74,79] (see Table 3).

#### 3.4.1. Interventions Including 2 Ps

##### Promotion and Placement

Seven studies examined the impact of interventions that used both promotion and placement strategies [46,53,54,55,56,57,58]. Two studies found positive effects [57,58], one found null effects [54], one found mixed (positive + null + negative) [55], and three found mixed effects (positive + null) [46,53,56].

An experimental design was used in only one study [46]. The intervention included a food marketing campaign (inclusive of food demonstrations, recipe cards, and an audio novella) featuring fruit and vegetable characters in tiendas [46]. Positive effects were found on daily fruit and vegetable intake but not variety [46].

Three studies employed quasi-experimental designs [53,54,58]. One intervention manipulated the in-store location of produce (i.e., moving pre-packaged produce near checkout lines), added shelf labels, and distributed recipe cards [58]. Another intervention focused on the effects of promoting meal bundles through in-store displays [54], while another examined the effects of pre-packaged produce packs moved to aisle endcaps packages [53]. One study found that shoppers who were exposed to the intervention were more likely to purchase produce [58], and another found that moving the pre-packaged produce near checkout lines increased healthy purchasing [53]. However, displaying meal bundles was ineffective in increasing healthy item sales [54]. One study used a pre-experimental design [57].

Two studies with time series designs addressed the effects of using behavioral nudges [56] and implementing a healthy food kiosk coupled with food sampling [55]. Results showed positive effects for healthy food sales when multiple behavioral nudges were implemented simultaneously [56] and when food sampling was combined with featured food kiosks [55]. Null and negative effects were found for healthy item sales when intervention tactics were isolated as well as among certain foods [55,56].

##### Promotion and Product

Five studies examined the impact of promotion and product interventions [47,48,49,50,51]. Three studies had mixed effects (positive + null) [47,49,51], one study had mixed effects (positive + null + negative) [50], and one had null effects [48].

All five studies utilized an experimental design [51,59,60,61,62] and included components related to increased stock of healthier items. Promotional strategies varied: One incorporated food demonstrations [47], one used social marketing campaigns [48], and all five used point-of-purchase promotions (e.g., taste testing, shelf labels, educational displays, food samples, and signage) [47,48,49,50,51]. All studies found at least one null effect on healthy food consumption and purchasing [47,48,49,50,51]. However, positive effects were shown in four of five studies as participants’ intent to purchase healthier foods increased with exposure to the interventions [47,49,50,51].

##### Promotion and Price

Only one study examined the effects of promotion and price and it used an experimental design [52]. The intervention examined the effects of healthy food consumption education and coupons with mixed effects (positive + null) on healthier purchases. Combining education and coupons was the most effective intervention group for increasing healthier purchases while null effects were largely observed for education and coupon only groups [52].

#### 3.4.2. Interventions Including 3 Ps

##### Promotion, Product, and Placement

Fifteen studies implemented interventions with promotion, product, and placement strategies [59,60,61,62,63,65,67,68,69,71,73,74,76,77,78]. Out of the 15, 2 had mixed effects (positive + negative) [68,78], 4 had mixed effects (positive + null) [59,60,61,62], 2 had mixed effects (positive + negative + null) [63,77], 3 had null effects [67,69,74], and 4 had positive effects [65,71,73,76].

Five studies were experimental [59,60,61,62,63]. The interventions included adding point-of-purchase promotions, changing the store structure and environment (e.g., adding a buffet bar or refrigerator, grouping products in a display), and altering the in-store location of products (e.g., multiple facings, prime placement, secondary placement, checkout aisle end-caps), and increased stocking of healthier products [59,60,61,62,63]. All five studies found mixed effects for improving the purchasing and consumption of healthy food. For example, Foster and colleagues (2014) implemented an intervention to increase the purchases of specific healthier foods through shelf tagging promotions and by altering the shelf placement of products [59]. In intervention stores, sales of 2% milk, whole milk, two targeted cereals, and one of three promoted frozen meals remained the same, while sales of skim milk, 1%, and two out of three frozen meals increased [59].

Four studies utilized quasi-experimental designs [65,67,68,69]. Two studies added point-of-purchase promotions, changed store structure and environment, altered in-store location, and increased stock of fresh produce [67,69]. Another study introduced healthier products to checkout lanes and added point-of-purchase promotions [68], and another changed store structure, increased media coverage about healthier choices, and offered in-store education sessions. Two studies found null effects on consumption and purchasing of fruits and vegetables [67,69], one found mixed effects (positive + negative) on consumer purchasing of healthy foods in healthy vs. standard checkout lanes [68], and one found positive effects of store owners’ perceptions of changes in sales of promoted healthy foods [65]. Of these four quasi-experimental studies, two interventions were Proyecto MercadoFRESCO [67,69]. Both studies found null effects, such that there were no significant differences in consumption of and dollars spent on fruit and vegetables [67,69].

Six studies in this category used a pre-experimental [71,73,74,76,77,78] design. Similar to previous studies, strategies added point-of-purchase promotions, changed store structure and environment, altered in-store location, and increased stock of fresh produce [71,74,76,77,78]; one study implemented these strategies and paired urban farms with corner stores such that corner stores sold products obtained from urban farms [73]. Three studies found positive effects on purchases, sales, consumption, and intent to purchase healthy food [71,73,76].

##### Promotion, Product, and Price

Three studies utilized promotion, product, and price marketing strategies [64,66,72]. One study found positive effects [66] and two found mixed effects (positive + null) [64,72].

Of the three studies, two studies used a quasi-experimental design [64,66]. Both were multifaceted interventions that included increased stocking of healthy foods, point-of-purchase promotions, and price reductions/incentive cards [64,66]. One of the studies found when shelf labels were consistently used (high fidelity), positive effects on sales of the promoted, healthy items were found [66]. The second quasi-experimental study found mixed effects (positive + null): shelf labels on healthy items led to participants purchasing more promoted foods but did not change consumption. However, the study authors did not observe changes in healthy food consumption. Finally, one study used a pre-experimental design [72], with mixed results.

##### Promotion, Placement, and Price

Two studies examined the effects of promotion, placement, and price strategies [70,75]. One study found null effects [70] and the other found positive effects [75]. Both studies used similar interventions, Plate It Up Kentucky Proud [75] and Plate It Up [70], which added point-of-purchase promotions, altered product placement, and offered coupons and discounts [70,75].

One study used a quasi-experimental design [70]. The results showed null effects on fruit and vegetable consumption. The study authors found no difference in the percent of food purchasing dollars spent on fruits and vegetables between control and intervention groups [70]. In addition, Liu and colleagues (2017) used a pre-experimental design [75] and found that recipe cards had a positive effect on customers’ purchases of recipe ingredients and increased consumption of fruits and vegetables [75].

#### 3.4.3. Intervention Including 4 Ps

Finally, only one study utilized all four Ps [79]. The study used an experimental, participatory design and found null effects for fruit and vegetable consumption. However, there was a significant decrease in the consumption of some unhealthy foods (e.g., chips) [79]. The intervention increased stocking of healthy foods, altered the in-store environment, added point-of-purchase promotions, and included discounts [79].

## 4. Discussion

This review, which examined the scope and impact of in-store marketing strategies related to healthy food sales, purchasing, and measures of diet, yields several important conclusions. One key finding of this recent review of literature is that both single- and multi-component interventions have become equally common focal points of research. Approaches provide evidence that increasing access to healthy food products in stores, particularly while utilizing promotion strategies, increases healthy food sales, purchasing, or improves dietary outcomes. While prior reviews found that positive outcomes were more common in studies utilizing multiple Ps [12,13], ours found more parity, even when considering the level of rigor applied to research designs and outcome measures. Overall, positive results were found in 27 of 30 single-component interventions as compared to 29 of 34 multi-component interventions, despite that multi-component interventions reported results related to a higher quantity of outcome measures.

Promotion efforts, including shelf labels, call out messages, and sampling products, continue to show promise as an important mechanism to improve purchasing. In-store promotion interventions are increasingly common, often with positive effects, either in combination with other approaches, or used alone. Previous reviews have found that older interventions, specifically those prior to 2008, were more likely to manipulate promotion, most often in single-component interventions [9,11]. In the more recent studies examined in this review, promotional interventions were frequently paired with placement and product strategies in multi-component interventions, for example including the coupling of a shelf labeling intervention with an end of aisle display, yielding positive effects.

Prior literature has identified multi-component interventions’ added complexities in deciphering effects of its individual components [4,11]. There are two reasons for this complexity. One is the layered nature of multi-component interventions which by definition result in activities such as taste-testing, coupled with an end-cap placement and a shelf tagging, which make it difficult to decipher how components work together or separately to influence purchasing. It is possible for example that similar effects could be seen from just a single-component intervention, rather than multiple, though such impacts are difficult to decipher. Future multi-component interventions should consider alternative research designs where elements of the intervention are incorporated at different times and in different combinations, and then removed and then incorporated again in order to understand collective and individual effects, such a 2 × 2 factorial design or an ABA design [80].

### Limitations and Future Directions

Of 64 studies reviewed, 24 in total (38%) were conducted without a control or comparison group. Only 14 of the 64 studies were experimental and included objective outcome measure data. The lack of a control group in more than one-third of studies displays the limitations of food environment research. Studies conducted with control groups, using store sales outcome data, and using rigorous dietary outcome measures are needed. Further research is also needed to better understand the individual and additive effects of multi-component interventions on outcomes like product sales.

The literature is limited in its ability to capture the extent to which increased healthy food sales results in overall less healthy food purchases. While several studies examine interventions in terms of specific product substitutions, for example by testing whether promoting a healthier item in a category results in changed sales in that product and a less healthy alternative (e.g., replacing higher fat popcorn with low-fat popcorn), few studies examine how targeted product sales relate to sales in other product categories (i.e., a spillover effect; e.g., increase in fruit sales associated with increase in low-fat dairy sales). Future research is needed to understand how increases in healthy food purchases do or do not serve to substitute for less healthy foods.

In addition to better understanding the marketing mechanisms that work best to shift purchasing, future research should examine the extent to which interventions yield sustained effects. Our review found that less than 20% of studies examined impacts beyond three months and only 4.5% considered impacts beyond one year.

It is unclear how the current COVID-19 context will continue to impact in-person food sales as compared to online sales and the extent to which product promotion and placement strategies can, or will, translate into online environments. Future work should seek to better understand how online food purchasing environments, including virtual supermarkets and real-world e-commerce platforms, can incorporate the four Ps to increase access to affordable foods.

## 5. Conclusions

Efforts to improve consumption and purchases of healthier foods in retail environments are diverse, even within the framework of the 4Ps. Considering these marketing strategies, this review found that promotion was the most commonly utilized strategy for single-component interventions, and manipulating promotion, placement, and product was the most common strategy used for multi-component intervention. In addition, interventions included in the review often employed pre-experimental or quasi-experimental research designs and relied more on self-report data rather than objective data. New research should implement interventions using rigorous designs and objective outcomes in order to advance the field. Further, given the large proportion of studies that implemented multi-component interventions, research is also needed to understand the individual and additive effects of approaches that use more than one of the 4Ps on objective sales outcomes, substitution effects of healthy food purchases, and the sustainability of impacts.

## Figures and Tables

**Table 1:** Study design characteristics for inclusion articles.

Factor		*n*	%
**Intervention Setting (*n* = 64) ^1^**			
	Supermarket	28	43.8%
	Corner Store (including tiendas, bodegas, and small food stores)	20	31.3%
	Grocery Store (including small markets, country stores, and local independent owned stores)	17	26.6%
	Convenience Store	6	9.4%
	Supercenter	2	3.1%
	Trading Post	2	3.1%
	Other (including large food retail stores and local food co-ops)	8	12.5%
**Research Design (*n* = 65) ^2^**			
	Experiment	23	35.4%
	Quasi-experiment	18	27.7%
	Pre-experiment	22	33.8%
	Time Series	2	3.1%
**Outcome Measures (*n* = 64) ^3^**			
Purchasing-Related Measures			
	Objective Store Sales Data	29	45.3%
	Objective Food Purchasing Data	6	9.4%
	Customer Receipts	5	7.8%
	Self-Report Purchases or Expenditures	25	39.1%
	Self-Report Store Sales	2	3.1%
	Self-Report Intent to Purchase	8	12.5%
Consumption-Related Measures			
	FFQ	2	3.1%
	24-h Dietary Recall	3	4.7%
	Veggie Meter^TM^ or other biometrics	1	1.6%
	Self-Report Intent to Eat	1	1.6%
	Other Self-Report Diet/Consumption Survey	12	18.8%
	Food Diary	0	--
**Duration of intervention (*n* = 64)**			
	1 min to 24 h	4	6.2%
	>24 h to 1 week	2	3.1%
	>1 week to 1 month	6	9.4%
	>1 month to 3 months	12	18.8%
	>4 months to 6 months	15	23.4%
	>6 months to 1 years	9	14.1%
	>1 year	15	23.4%
	Not reported	1	1.6%
**Duration of follow-up (*n* = 66) ^4^**			
	No follow-up (i.e., collected data while intervention was being implemented)	24	36.4%
	Immediately following the intervention	15	22.7%
	Not reported	4	6.1%
	≤1 week	1	1.5%
	>1 week to 1 month	6	9.1%
	>1 month to 3 months	3	4.5%
	>3 months to 1 year	10	15.2%
	>1 year	3	4.5%
**Participant sample size at follow-up (*n* = 64)**			
	Not reported or indeterminate	17	26.6%
	≤100	10	15.6%
	101–500	23	35.9%
	501–1000	8	12.5%
	>1000	6	9.4%
**Store sample size at follow-up (*n* = 64)**			
	Not reported, not applicable, or indeterminate	21	32.8%
	≤2	13	20.3%
	3–10	19	29.7%
	11–20	4	6.3%
	>20	7	10.9%
**Participant response rate at follow-up (*n* = 64)**			
	Not reported, not applicable, or indeterminate	41	64.1%
	<50%	3	4.7%
	50% to 75%	12	18.8%
	76% to 90%	4	6.2%
	>90%	4	6.2%

^1^ Percentages do not add up to 100 because multiple intervention settings were used in some studies; ^2^ One intervention had two study designs; ^3^ Percentages do not add up to 100 because multiple outcomes were used in some studies; ^4^ Two interventions have different follow-up periods for difference stores.

**Table 2:** Summary of included single-component interventions: Design, focus, and effects.

References	Study Design	Price	Prod	Prom	Place	Other	Intervention Name & Length	Outcome Measures	Food Categories	Effect
Milliron et al., A point-of-purchase intervention featuring in-person supermarket education affects healthful food purchases. (2012) [17].	EXP			P			*EatSmart* program, 4 months	Customer receipts [+] Objective food purchasing data (digital photographs) [+, null]	Fruits and vegetables (fresh)	Mixed^^
Geliebter et al., Supermarket discounts of low-energy density foods: Effects on purchasing, food intake, and body weight. (2013) [33].	EXP	P					Supermarket discounts intervention, 8 weeks	Objective store sales data [+] 24-h dietary recall [+]	Fruits and vegetables	Positive
Kiesel & Villas-Boas, Can information costs affect consumer choice? nutritional labels in a supermarket experiment. (2013) [22].	EXP			P			Nutritional labelling intervention, 4 weeks	Objective store sales data [+, −, null]	Salty snacks (popcorn)	Mixed^^^
Tal & Wansink, An apple a day brings more apples your way: Healthy samples prime healthier choices. (2015) [34].	EXP			P			Healthy and indulgent food samples, Not reported	Objective food purchasing data (bag checks) [+]	Fruits and vegetables	Positive
Bernales-Korins et al., Psychosocial influences on fruit and vegetable intake following a NYC supermarket discount. (2017) [18].	EXP	P					Supermarket discount intervention, 8 weeks	Objective store sales data [+, null] 24-h dietary recall [+, null]	Fruits and vegetables	Mixed^^
Liu et al., The Sales Impact of Featuring Healthy Foods, Indulgent Foods, or Both: Findings from a Large-Scale Retail Field Study. (2018) [23].	EXP				P		Endcap food displays, 2 months	Objective store sales data [+, −, null]	Nuts Protein (protein bars) Salty snacks (granola bars) Sweets (candy and cookies)	Mixed^^^
Palacios et al., Effectiveness of the Nutritional App “MyNutriCart” on Food Choices Related to Purchase and Dietary Behavior: A Pilot Randomized Controlled Trial. (2018) [24].	EXP			P			“MyNutricart” smartphone application, 8 weeks	Customer receipts [+} FFQ [+, null] 24-h dietary recall [+, −]	Beverages (100% juice) Dairy Fruits and vegetables Grains Legumes Proteins (meats) Salty Snacks Sweets	Mixed^^^
Biswas et al., Sounds like a healthy retail atmospheric strategy: effects of ambient music and background noise on food sales. (2019) [16].	EXP					P	Retail atmosphere intervention, 2 weekdays	Objective store sales data [+, −]	Dairy (yogurt and eggs) Fruits and vegetables Grains (bread) Protein (ham and pork) Salty snacks (chips) Soup Sweets (candy, cakes, and cookies)	Mixed^
Biswas & Szocs, The smell of healthy choices: Cross-modal sensory compensation effects of ambient scent on food purchases. (2019) [35].	EXP					P	Ambient scent 1 h	Customer receipts [+]	Dairy (milk) Fruits and vegetables (including fried potatoes) Grains (crackers) Protein (chicken) Salty snacks (chips) Sweets (Rice Krispy treats and fruit cobbler)	Positive
Berning et al., Do positive nutrition shelf labels affect consumer behavior? findings from a field experiment with scanner data. (2011) [30].	QE			P			Positive nutrition shelf labels, 4 weeks	Objective store sales data [−]	Salty snack (popcorn)	Negative
Rahkovsky et al., Effects of the Guiding Stars Program on purchases of ready-to-eat cereals with different nutritional attributes. (2013) [36].	QE			P			Guiding Stars, 20 months	Objective store sales data [+]	Grains (cereal)	Positive
Nikolova & Inman, Healthy choice: The effect of simplified point-of-sale nutritional information on consumer food choice behavior. (2015) [37].	QE			P			NuVal Nutritional Scoring System, 6 months	Objective store sales data [+]	Dairy (yogurt and ice cream) Frozen meals (pizza) Salty snacks (granola bars) Sauces and dressing Soup	Positive
Schultz & Litchfield, Evaluation of traditional and technology-based grocery store nutrition education. (2016) [38].	QE			P			Aisle demonstrations and technology-based education treatments, 4 months	Other self-report diet/consumption survey [+]	Fruits and vegetables Grains (whole grains) Protein (lean meats and seafood)	Positive
Finnell et al., 1% low-fat milk has perks!: An evaluation of a social marketing intervention. (2016) [25].	QE			P			1% Low-Fat Milk Has Perks!, 12 weeks	Objective store sales data [+, −, null]	Dairy (milk)	Mixed^^^
Zhen & Zheng, The impact of NuVal shelf nutrition labels on food purchase. (2017) [39].	QE			P			NuVal Nutritional Scoring System, 4 months	Objective store sales data [+]	Dairy [yogurt]	Positive
Bachman & Arigo, Reported influences on restaurant-type food selection decision making in a grocery store chain. (2018) [26].	QE			P			Calorie labelling intervention, 1 month	Self-report purchases [+, −, null]	Deli and bakery prepared foods	Mixed^^^
Jilcott Pitts et al., One-year follow-up examination of the impact of the North Carolina Healthy Food Small Retailer Program on healthy food availability, purchases, and consumption. (2018) [31].	QE			P			North Carolina Healthy Food Small Retailer Program, 6 months	Objective food purchasing data (bag checks) [null] Veggie Meter^TM^ [null] Other self-report diet/consumption survey [null]	Beverages (SSBs) Fruits and vegetables (fresh, canned, and frozen)	Null
Jetter & Cassady, Increasing fresh fruit and vegetable availability in a low-income neighborhood convenience store: A pilot study. (2010) [40].	PE		P				Produce availability intervention, 7 months	Objective store sales data [+]	Fruits and vegetables (fresh)	Positive
Sutherland et al., Guiding Stars: The effect of a nutrition navigation program on consumer purchases at the supermarket. (2010) [41].	PE			P			Guiding Stars, 2 years	Objective store sales data [+]	Grains (cereal)	Positive
Bangia & Palmer-Keenan, Grocery store podcast about omega-3 fatty acids influences shopping behaviors: A pilot study. (2014) [42].	PE			P			Podcast, 5 min	Self-report purchases [+] Self-report intent to purchase [+]	Protein (N-3-rich foods)	Positive
Cawley et al., The impact of a supermarket nutrition rating system on purchases of nutritious and less nutritious foods. (2015) [19].	PE			P			Guiding Stars, 15 months	Objective store sales data [+, null]	All food categories	Mixed^^
Weiss et al., Consumer taste tests and milk preference in low-income, urban supermarkets. (2015) [43].	PE			P			Healthy Retail Solutions milk taste testing intervention, 2 min >>note same study as Foster et al., 2014	Self-report intent to purchase [+]	Dairy (milk)	Positive
Bangia et al., A point-of-purchase intervention using grocery store tour podcasts about omega-3s increases long-term purchases of omega-3-rich food items. (2017) [20].	PE			P			Podcast, 22 min	Objective store sales data [+] Self-report intent to purchase [null]	Protein (N-3-rich foods)	Mixed^^
Lopez et al., Development and evaluation of a nutritional smartphone application for making smart and healthy choices in grocery shopping. (2017) [44].	PE			P			Smartphone nutrition application, 8 weeks	Self-report purchases [+]	Fruits and vegetables	Positive
Gustafson & Zeballos, The effect of ingredient-specific calorie information on calories ordered. (2018) [27].	PE			P			Ingredient specific calorie labeling, 8 months	Customer receipts [+, −, null]	Dairy (cheese) Grains (bread) Protein (deli meats) Vegetables	Mixed^^^
Finkelstein et al., Identifying the effect of shelf nutrition labels on consumer purchases: results of a natural experiment and consumer survey. (2018) [21].	PE			P			NuVal Nutritional Scoring System, 138 weeks	Objective store sales data [+, null]	Dairy (miscellaneous dairy, milk, and yogurt)	Mixed^^
Gustafson et al., Community-wide efforts to improve the consumer food environment and physical activity resources in rural Kentucky. (2019) [45].	PE			P			Plate it Up Kentucky Proud (PIUKP), 12 months	Other self-report diet/consumption survey [+]	Fruits and vegetables	Positive
Melo et al., Does point-of-sale nutrition information improve the nutritional quality of food choices? (2019) [28].	PE			P			NuVal Nutritional Scoring System, 14 months	Objective store sales data [+, −, null]	Dairy (yogurt) Frozen meals Grains (cereal)	Mixed^^^
Privitera et al., Impact of price elasticity on the healthfulness of food choices by gender. (2019) [29].	PE	P					Price elasticity conditions, 1 week	Self-report purchases [+, null] Self-report expenditures [+, −, null]	Fruits and vegetables	Mixed^^^
Sutton et al., Healthy food marketing and purchases of fruits and vegetables in large grocery stores. (2019) [32].	PE			P			Nutrition Education and Obesity Prevention program, 5 months	Self-report purchases [null]	Fruits and vegetables	Null

EXP = experimental; QE = quasi-experiment; PE = pre-experiment; Mixed^ = positive + negative; Mixed^^ = positive + null; Mixed^^^ = positive + null + negative; ‘P’ indicates that the intervention utilized this marketing approach.

**Table 3:** Summary of included multi-component interventions: Design, focus, and effects.

References	Study Design	Price	Prod	Prom	Place	Other	Intervention Name & Length	Outcome Measures	Food Categories	Effect
Ayala et al., Efficacy of a store-based environmental change intervention compared with a delayed treatment control condition on store customers’ intake of fruits and vegetables. (2013). [46]	EXP			P	P		Food marketing campaign 4 months	Other self-report diet/consumption survey [+, null]	Fruits and vegetables (fresh, canned, and frozen)	Mixed^^
Gittelsohn et al., A food store-based environmental intervention is associated with reduced BMI and improved psychosocial factors and food-related behaviors on the Navajo nation. (2013) [47].	EXP		P	P			Navajo Healthy Stores 14 months	Self-report intent to purchase [+, null] Self-report intent to eat [+, null] Self-report purchases [+, null]	41 healthy food items and 12 unhealthy food items: Beverages (soda) Grains (whole-wheat bread)	Mixed^^
Foster et al., Placement and promotion strategies to increase sales of healthier products in supermarkets in low-income, ethnically diverse neighborhoods: A randomized controlled trial. (2014) [59].	EXP		P	P	P		In-store marketing strategies intervention 6 months	Objective store sales data [+, null]	Beverages Dairy (milk) Frozen meals Grains (cereal)	Mixed^^
Lent et al., A randomized controlled study of a healthy corner store initiative on the purchases of urban, low-income youth. (2014) [48].	EXP		P	P			Snackin’ Fresh Intervention 2 years	Self-report purchases [null]	Beverages Grains (bread) Protein (deli meat) Salty snacks (chips) Sweets (candy)	Null
Martinez-Donate et al., Evaluation of a pilot healthy eating intervention in restaurants and food stores of a rural community: A randomized community trial. (2015) [49].	EXP		P	P			Waupaca Eating Smart 10 months	Self-report purchases [+, null]	Fruits and vegetables	Mixed^^
Shin et al., Impact of Baltimore healthy eating zones: An environmental intervention to improve diet among African American youth. (2015) [50].	EXP		P	P			Baltimore Healthy Eating Zones 8 months	Self-report purchases [+, −, null] Self-report intent to purchase [+, −]	Fruits and vegetables Grains (whole wheat bread and cereal) Salty snacks (trail mix) Nuts and seeds	Mixed^^^
Gittelsohn et al., The impact of a multi-level multi-component childhood obesity prevention intervention on healthy food availability, sales, and purchasing in a low-income urban area. (2017) [51].	EXP		P	P			B’more Healthy Communities for Kids (BHCK), 2 years	Objective store sales data [+, null] Self-report purchases [null]	Beverages (soda, energy drinks, water, 100% fruit juice, and unsweetened tea) Dairy (milk and yogurt) Fruits and vegetables (fresh and dried) Grains (cereals and bread) Nuts and seeds Protein (canned tuna and dried beans) Salty snacks (pretzels, and chips)	Mixed^^
Thorndike et al., Choice architecture to promote fruit and vegetable purchases by families participating in the special supplemental program for women, infants, and children (WIC): Randomized corner store pilot study. (2017) [60].	EXP		P	P	P		Choice architecture intervention 5 months	Objective store sales data [+] Self-report purchases [+, null]	Fruits and vegetables	Mixed^^
Banerjee & Nayak, Believe it or not: Health education works. (2018) [52].	EXP	P		P			Healthy food consumption education 2 weeks	Objective store sales data [+, null]	Fruits and vegetables (fresh) Grains (whole grain)	Mixed^^
Trude et al., A multilevel, multicomponent childhood obesity prevention group-randomized controlled trial improves healthier food purchasing and reduces sweet-snack consumption among low-income African-American youth. (2018) [61].	EXP		P	P	P		BHCK 14 months	Self-report purchases [+, null]	Dairy (string cheese, yogurt, and ice cream) Fruits and vegetables (fresh and canned) Grains (cereals) Salty snacks (popcorn, chips, and pretzels) Sweets (candy, cookies, cakes, pies, and donuts)	Mixed^^
Bird Jernigan et al., A Healthy Retail Intervention in Native American Convenience Stores: The THRIVE Community-Based Participatory Research Study. (2019) [79].	EXP	P	P	P	P		Tribal Health and Resilience in Vulnerable Environments study 9–12 months	Other self-report diet/consumption survey [null]	Fruits and vegetables (including fried potatoes) Protein (meat) Salty snacks (chips)	Null
Trude et al., The impact of a multilevel childhood obesity prevention intervention on healthful food acquisition, preparation, and fruit and vegetable consumption on African-American adult caregivers. (2019) [62].	EXP		P	P	P		BHCK 14 months	Other self-report diet/consumption survey [+, null] Self-report purchases [null]	Fruits and vegetables	Mixed^^
Wensel et al., B’more healthy corner stores for moms and kids: Identifying optimal behavioral economic strategies to increase WIC redemptions in small urban corner stores. (2019) [63].	EXP		P	P	P		B’more Healthy Corner Stores 4 Moms and Kids 1 year	Objective store sales data [+, −, null]	Beverages (juice) Dairy Fruits and vegetables (fresh) Grain (miscellaneous grains and cereal) Infant foods and formula Protein	Mixed^^^
Gittelsohn et al., An urban food store intervention positively affects food-related psychosocial variables and food behaviors. (2010) [64].	QE	P	P	P			Baltimore Healthy Stores 10 weeks	FFQ [+, null] Self-report intent to purchase [+]	26 healthy food items: Beverages Dairy (milk) Fruits and vegetables Grains (cereal and pretzels)	Mixed^^
Steeves et al., A rural small food store pilot intervention creates trends toward improved healthy food availability. (2015) [65].	QE		P	P	P		Maryland Healthy Stores 4 months	Self-report store sales data [+]	Dairy (milk and cheese) Fruits and vegetables Grains (whole wheat bread) Salty snacks (baked chips)	Positive
Surkan et al., Eat Right-Live Well! supermarket intervention impact on sales of healthy foods in a low-income neighborhood. (2016) [66].	QE	P	P	P			Eat Right-Live Well! (ERLW), 3 months	Objective store sales data [+]	Beverages (sugar-sweetened beverages [SSBs]) Dairy Fruits and vegetables Grains Salty snacks Sweets	Positive
Ortega et al., Substantial improvements not seen in health behaviors following corner store conversions in two Latino food swamps. (2016) [67].	QE		P	P	P		Proyecto Mercado FRESCO 2 years	Self-report expenditures [null] Other self-report diet/consumption survey [null]	Fruits and vegetables	Null
Adjoian et al., Healthy checkout lines: A study in urban supermarkets. (2017) [68].	QE		P	P	P		Healthy checkout lanes 2 weeks	Objective food purchasing data (checkout line observations) [+, −]	Beverages (water and seltzer) Fruits (fresh and dried) Nuts and seeds Salty snacks (granola bars, trail mix, and chips)	Mixed^
Albert et al., A corner store intervention to improve access to fruits and vegetables in two Latino communities. (2017) [69].	QE		P	P	P		Proyecto MercadoFRESCO 3.5 years	Self-report purchases [null] Other self-report diet/consumption survey [null] Self-report expenditure [null]	Fruits and vegetables (fresh, canned, and frozen)	Null
Payne & Niculescu, Can healthy checkout end-caps improve targeted fruit and vegetable purchases? evidence from grocery and SNAP participant purchases. (2018) [53].	QE			P	P		Healthy checkout aisle end-caps 1 month	Objective store sales data [+, null]	Fruits and vegetables	Mixed^^
Gustafson et al., The association between the “Plate it Up Kentucky” supermarket intervention and changes in grocery shopping practices among rural residents. (2019) [70].	QE	P		P	P		Plate it Up Kentucky 3 months	Customer receipts [null] Other self-report diet/consumption survey [null]	Beverages (SSBs) Fruits and vegetables	Null
Moran et al., Make It Fresh, for Less! A supermarket meal bundling and electronic reminder intervention to promote healthy purchases among families with children. (2019) [54].	Study 1: QEStudy 2:EXP			P	P		Study 1: Make it Fresh for Less! Study 2: Electronic reminders 16 weeks	Objective store sales data [null] Self-report purchases [null]	Various meal recipe ingredients with and without fruits and vegetables	Null
Holmes et al., Effect of a grocery store intervention on sales of nutritious foods to youth and their families. (2012) [55].	Time series			P	P		Healthy Kids Campaign, 12 weeks	Objective food purchasing data (cart checks) [+] Objective store sales data [+, −, null]	Dairy (milk and string cheese) Fruits and vegetables (fresh) Grains (whole wheat bagels) Nuts and seeds (sunflower seeds) Salty snacks (chips)	Mixed^^^
Chapman et al., Evaluation of three behavioural economics ‘nudges’ on grocery and convenience store sales of promoted nutritious foods. (2019) [56].	Time series			P	P		Behavioral economic nudges 6 months	Objective store sales data [+, null] Self-report intent to purchase [+]	Fruits and vegetables (fresh) Salty snack (granola bars)	Mixed^^
Dannefer et al., Healthy bodegas: Increasing and promoting healthy foods at corner stores in New York City. (2012) [71].	PE		P	P	P		Healthy Bodegas Initiative 5 months	Self-report purchases [+] Self-report store sales data [+]	Dairy (milk) Fruits and vegetables (fresh and canned) Grain (whole-grain bread) Salty Snacks	Positive
Paek et al., Assessment of a healthy corner store program (FIT store) in low-income, urban, and ethnically diverse neighborhoods in Michigan. (2014) [72].	PE	P	P	P			Fit Store Program 6 months	Other self-report diet/consumption survey [+, null] Self-report purchases [+]	Beverages (100% fruit juice) Dairy (low-fat milk) Fruits and vegetables (fresh) Grains (whole grain bread and brown rice) Salty snacks Nuts and seeds Legumes	Mixed^^
Gudzune et al., Increasing access to fresh produce by pairing urban farms with corner stores: a case study in a low-income urban setting. (2015) [73].	PE		P	P	P		Farmers and corner store intervention, 9 weeks	Objective store sales data [+]	Fruits and vegetables (fresh)	Positive
Lawman et al., Changes in quantity, spending, and nutritional characteristics of adult, adolescent and child urban corner store purchases after an environmental intervention. (2015) [74].	PE		P	P	P		Healthy Corner Store Initiative, 12 months	Objective food purchasing data (bag checks) [null] Self-report expenditure [null]	Beverages Grains (bread) Protein (deli meat) Salty Snacks (chips) Sweets (candy)	Null
Davis et al., Employee and customer reactions to a healthy in-store marketing intervention in supermarkets. (2016) [57].	PE			P	P		Healthy in-store marketing intervention 6 months	Self-report purchases [+]	Dairy (milk) Frozen meals Grains	Positive
Liu et al., Marketing strategies to encourage rural residents of high-obesity counties to buy fruits and vegetables in grocery stores. (2017) [75].	PE	P		P	P		PIUKP 4 months	Other self-report diet/consumption survey [+]	Fruits and vegetables	Positive
Rushakoff et al., Evaluation of Healthy2Go: a country store transformation project to improve the food environment and consumer choices in Appalachian Kentucky. (2017) [76].	PE		P	P	P		Healthy2Go 18 months	Self-report purchases [+] Self-report intent to purchase [+] Other self-report diet/consumption survey [+]	Beverages (water, soda, and 100% juice) Dairy (milk) Fruits and vegetables (fresh, canned, and frozen) Grains Salty snacks (chips) Nuts and seeds	Positive
Woodward-Lopez et al., Changes in consumer purchases in stores participating in an obesity prevention initiative. (2018) [77].	PE		P	P	P		Kaiser Permanente Healthy Eating and Active Living, 1 year (Zones 1 and 3) and 3 years (Zone 2)	Objective store sales data [+, −, null] Self-report purchases [+]	Beverages (SSBs)Fruits and vegetables Salty snacks (chips) Sweets (candy)	Mixed^^^
MacKenzie et al., Healthy Stores Initiative Associated with Produce Purchasing on Navajo Nation. (2019) [58].	PE			P	P		Healthy Navajo Stores Initiative 1 year	Self-report purchases [+]	Fruits and vegetables (fresh and frozen)	Positive
Paluta et al., Evaluating the impact of a healthy corner store initiative on food access domains. (2019) [78].	PE		P	P	P		Fresh Foods Here 10 months	Objective store sales data [+]	Healthy items which were classified as low sodium, low fat, and low sugar	Mixed^

EXP = experimental; QE = quasi-experiment; PE = pre-experiment; Mixed^ = positive + negative; Mixed^^ = positive + null; Mixed^^^ = positive + null + negative; ‘P’ indicates that the intervention utilized this marketing approach.
